# Updates implemented in version 4 of the GlyCosmos Glycoscience Portal

**DOI:** 10.1007/s00216-024-05692-0

**Published:** 2024-12-17

**Authors:** Sunmyoung Lee, Tamiko Ono, Shiota Masaaki, Akihiro Fujita, Masaaki Matsubara, Achille Zappa, Issaku Yamada, Kiyoko F. Aoki-Kinoshita

**Affiliations:** 1 Glycan and Life Systems Integration Center (GaLSIC), Soka University, Hachioji, Tokyo Japan; 2 Graduate School of Science and Engineering, Soka University, Hachioji, Tokyo Japan; 3 Institute for Glyco-Core Research, Nagoya University, Nagoya, Japan; 4 The Noguchi Institute, Tokyo, Japan

**Keywords:** Glycan-related genes, Glycan-related disease, Glycans, Repository, Data integration

## Abstract

Glycosylation, characterized by its complexity and diversity, is a common system across all domains of life. The glycosylation of proteins or lipids imparts them with structural and functional roles, ranging from development to infectious or Mendelian disease. The high-throughput-based omics data has revealed that glycans are involved in important cellular processes. Comprehensive knowledge of glycosylation has contributed not only to the fundamental concepts in glycoscience but also to its applications, including the development of molecular markers for diagnosis and therapeutic tools for treating diseases. The GlyCosmos Glycoscience Portal (GlyCosmos) has undergone significant updates to better support the scientific community in studying glycosylation-related phenomena. Key enhancements include the integration of expanded datasets linking glycans to other omics fields, improved tools for glycan structure prediction and analysis, and upgraded visualization capabilities to streamline data interpretation. A strengthened focus on data standardization has also been introduced, fostering interoperability between glycoscience resources and external databases. Since its release in 2019, the portal has seen a fivefold increase in user engagement, reflecting its growing relevance. These recent advancements aim to provide researchers with a more comprehensive and user-friendly platform, enabling deeper insights into glycan roles in cellular processes and disease mechanisms. GlyCosmos will continue to evolve, prioritizing community needs and advancing the integration of glycoscience with broader biological and biomedical research.

**Graphical Abstract:**

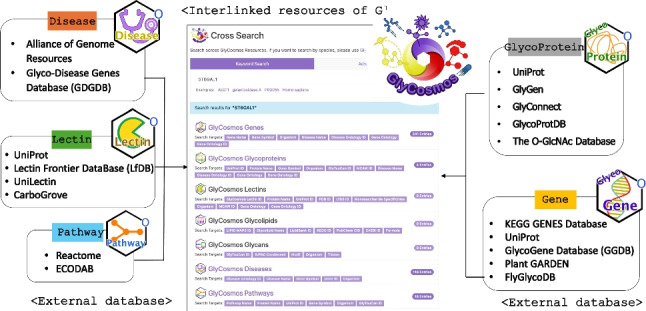

## Introduction

Despite the complexity of glycan structure features (e.g., alpha or beta configuration) and their branched structure, with advances in analysis techniques such as mass spectrometry (MS) and high-performance liquid chromatography (HPLC), ever-growing volumes of glycans found in all living organisms have been cataloged in various databases. Along with the diversity of glycan structures, numerous studies over the past decades have revealed the functions of glycan-binding proteins or glycoconjugates in many cellular activities such as cell-to-cell or immune-mediated communication [1, 2], cellular development [3], host–pathogen interaction [4], and numerous diseases, including cancer, infectious disease, autoimmune disease, and genetic disorders [5]. In addition, recent studies using artificial intelligence (AI) have been introducing new predictive information regarding glycosylation processes [6] and glycan interactions between host and pathogen [7].

The GlyCosmos Portal is a knowledge platform that provides not only glycan-related information but also insights into the knowledge required to understand the role of glycans [8]. Comprehensive data is essential to better understand the roles of glycans in various cellular processes because biological data are not independent. Integrated data reveal the relationships between biomolecules, which is essential for designing experiments for further investigation and gaining new insights. However, biological data that features complexity and heterogeneity has posed a challenge for data integration because a significant amount of data is still stored in individual databases despite the rapid growth of omics data, including genomics, proteomics, metabolomics, transcriptomics, and interactomics. In particular, inconsistencies or obscurities of terms give rise to inaccurate descriptions of resources for both humans and computers, making it difficult to share or integrate data between various databases and research groups.

Based on this background, we have strived to communicate and collaborate with experimental biologists and bioinformaticians to keep pace with new experimental results from glycomics and other life science databases, as well as to integrate the burgeoning data generated from other omics fields. These integrations are systematically planned and accomplished in compliance with the FAIR data principles, which stand for findable, accessible, interoperable, and reusable, for the sake of reuse and sharing of data. Pursuing data integration in a standard manner, the GlyCosmos Portal is constructed based on Semantic Web technologies, including RDF, SPARQL, and ontologies. This allows glycan-related data to be integrated in a formal format, where the resources are defined in a standardized, machine-readable, and computer-understandable manner [9]. The GlyCosmos Portal is organized into subcategories to effectively manage the copious glycan-related data: The Submission section contains four repository systems for glycan structures, glycoconjugates, and raw data for mass spectrometry; the Resources section contains genes, glycoproteins, glycolipids, lectins, pathways, and diseases; and the Tools section provides various tools, including a glycan drawing tool that facilitates the conversion between text and graphic forms of glycans, and several analytical tools, including GlycoMaple [10], which uses enzyme expression information entered to predict glycan profiles, and GlycomeAtlas (https://glycosmos.org/glycomeatlas), which displays the distribution of glycans in the tissues of humans, mice, and zebrafish. In the latest updated version 4 of GlyCosmos described here, the GlycoMaple tool has been updated to provide support for the investigation of glycan-related pathways in mice.

The biological knowledge built on standardized representations, particularly ontologies, will enable computers to easily integrate and exploit knowledge [11]. Our effort to integrate data across disciplines enables users unfamiliar with glycans to easily access glycan-related information. An example of such data integration is PubChem, a representative database that provides detailed information on chemical structures and their biological activities in the fields of chemistry and biology [12]. However, one of the challenges to integration is the ambiguity and abundance of synonyms in the names of biomolecules, which makes it difficult to integrate various sources. The GlyCosmos project has made an effort to explicitly explain resources by mapping them to vocabularies provided by domain-specific ontologies, including Disease Ontology (DO) [13], Gene Ontology (GO) [14], Uber-anatomy Ontology (UBERON) [15], and DDBJ Taxonomy. Here, we report the updates to the GlyCosmos Portal, including more comprehensive resources through integration with omics data, a new repository for submitting glycoconjugates, a new data model schema to enhance interconnectivity, and an improved search interface for efficient retrieval of information from large datasets. Moreover, we will also indicate where inferencing has been used, based on the Semantic Web technologies used in GlyCosmos, which have aided in supplementing the data in this portal.

## Results

### New RDF schema

**Fig. 1:**
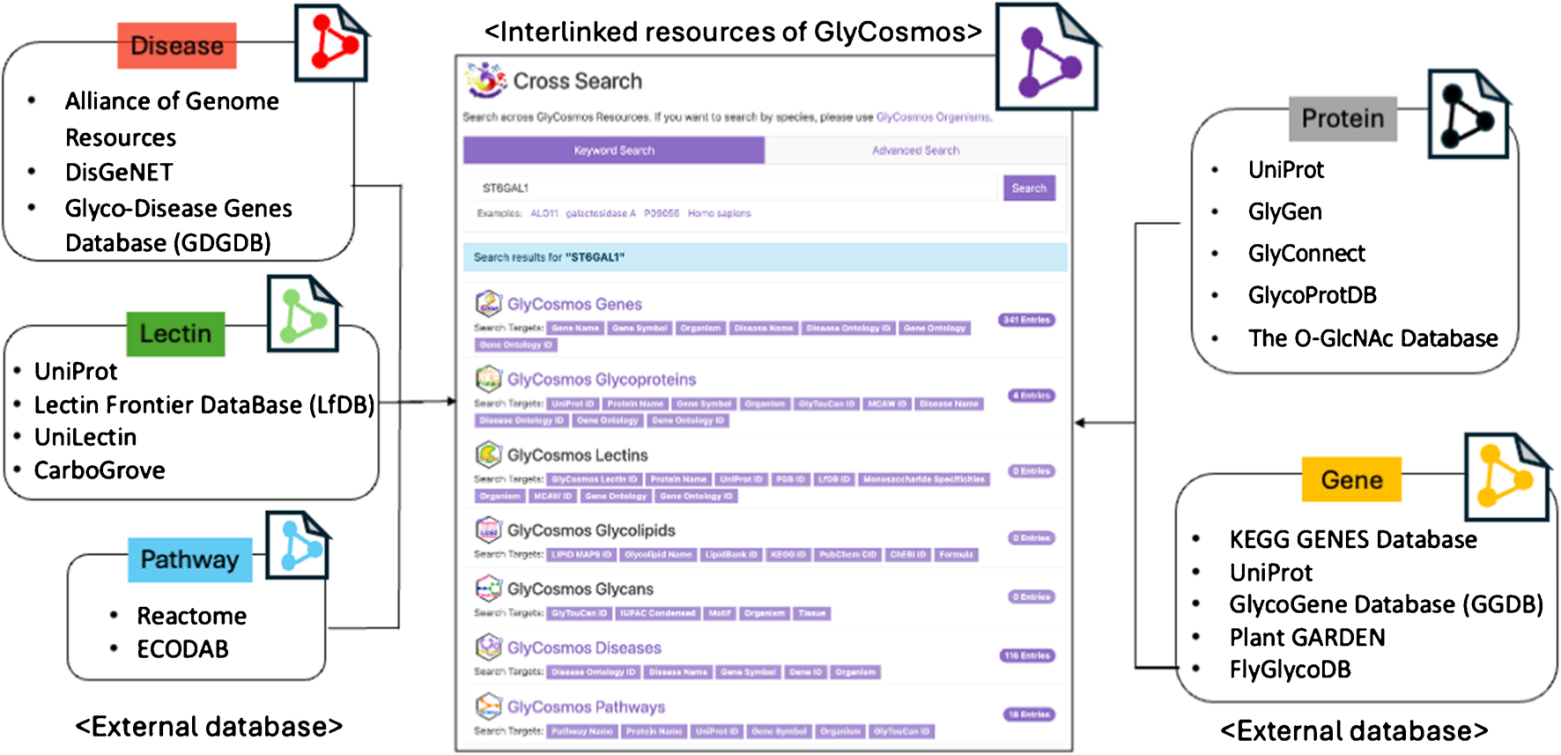
Data in previous versions of GlyCosmos possessed distinct RDF schemas, with the data structure designed according to the specific goals of each database. The unified RDF schema in the new version links all resources internally and semantically, enabling users to quickly and easily inspect glycan-related data through a comprehensive dataset

One of the main goals of GlyCosmos is to describe resources in a standard format to facilitate data integration. We have applied semantic standards, including RDF, SPARQL, and ontologies, to describe the data of GlyCosmos. As a result, the data is organized into semantically interconnected graph data that can describe the relationships between data within datasets. This allows for the easy addition and modification of data within the existing RDF graph, which provides flexibility for data integration and is appropriate for the dynamic nature of biological data. Another benefit of the RDFized data is that it supports sophisticated queries across multiple databases using a federated search technique. However, the data imported from an external database possesses its own RDF graph schema or data structure, so we have to handle it in accordance with its independent schema. This has led to an increase in the intricacy of queries and a time delay in obtaining results from SPARQL queries due to the need to traverse through the various graphs that contain the required resources. Figure 1 illustrates that glycan-related data, which is distributed across many databases that are devoted to specific resources, can be systematically organized and linked via our unified RDF schema, enabling users to easily inspect all the relevant information while navigating the interconnected dataset. Resources, encompassing glycogenes, glycoproteins, and glycan-associated disorders, can be queried using many terms within the purple box, including standard name, identifier number, several synonyms, species, and others. For example, inputting the gene name ST6GAL1 will provide all resources containing the keyword and display the related keywords available for search. Clicking the resource will transfer us to specific sites for each relevant resource, containing information on the gene, protein, and associated diseases.

**Fig. 2:**
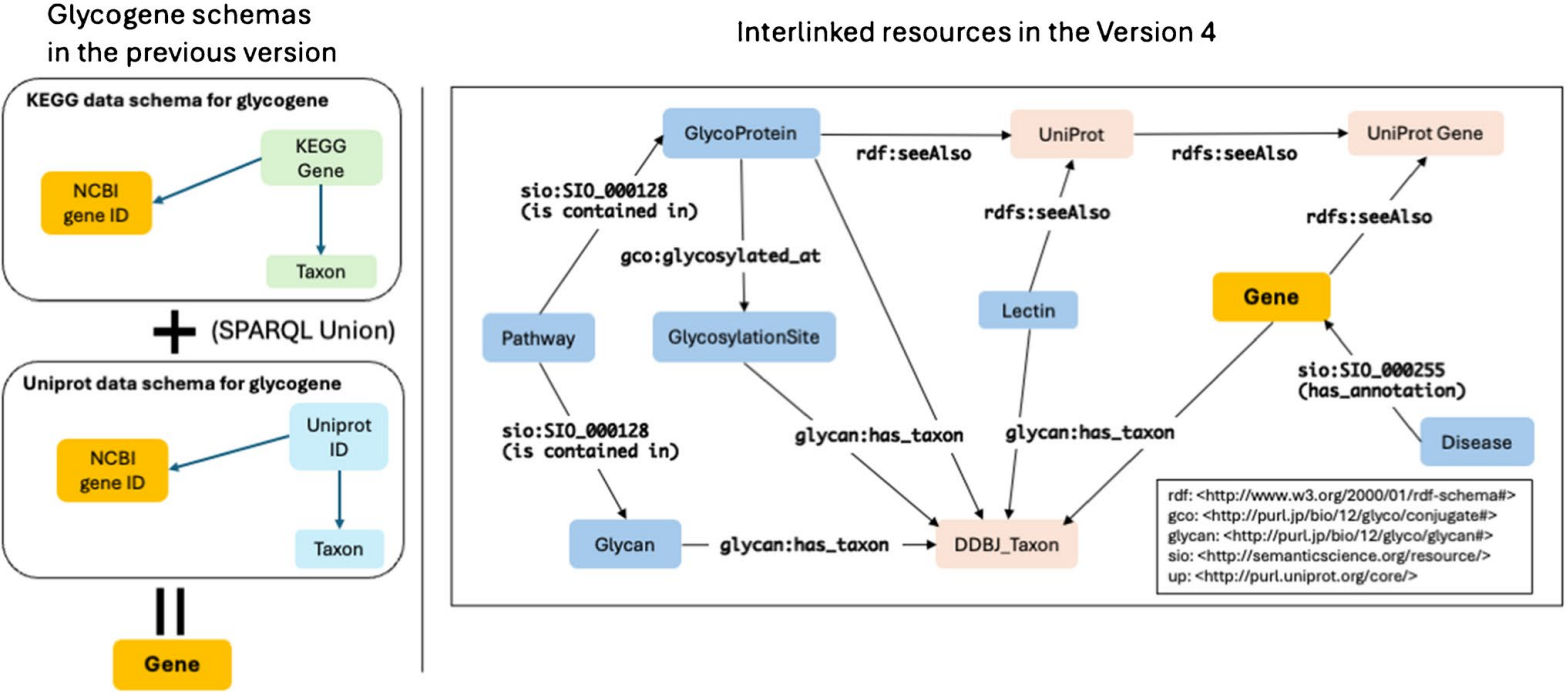
Representative resources and their relationships in a new RDF schema. Blue and pink in the left box represent GlyCosmos and external resources, respectively. The Programmatic Access in the menu of the portal homepage provides a more detailed schema of how resources are connected to each other within the knowledge graph of the GlyCosmos (https://glycosmos.org/programmatic). Each resource in the old version had its own RDF schema, which retarded time for retrieving because of the complex SPARQL queries

With a new RDF schema to efficiently manage the updated data and explicitly describe the relationships among resource data within GlyCosmos (Fig. 2), resources that came from a variety of datasets are fully interconnected, and search performance is enhanced in GlyCosmos version 4.0. The detailed representation of the RDF schema and ontologies is identified at the GlyCosmos Programmatic Access page (https://glycosmos.org/programmatic). Also, data from each resource page is accessible as an RDF file in turtle format and a CSV file format for download.

Ontologies play an essential role in dealing with confusion that is caused by uncertainty of vocabulary, as the terms used in life science, including biomolecules, anatomy, disease, and phenotypes, are highly complex and have multiple synonyms for the same concepts. A new RDF model has been developed to use existing controlled vocabularies and ontologies, such as GlycoRDF [16], GlycoCoO [17], and the Semanticscience Integrated Ontology (SIO) [18], etc. In particular, the adaptation of ontologies for species and genes, such as NCBI Taxonomy and the GO ontology, enables us to implement inference-based analysis. For instance, in GO annotations, the gene function or gene products are organized into hierarchical relationships, using the term *rdfs:subClassOf*. By leveraging ontology-defined inferred relationships, we were able to reveal the hidden connection between glycan-related genes and their associated gene annotations. This enables the Genes resource entry page to provide useful tables that enable users to search Genes based on hierarchically categorized gene functions. Moreover, the result datasets can be narrowed down from a total of 8156 gene datasets by entering metadata such as gene names or species names into the dialog box that provides filtering search. We also use *rdfs:seeAlso* properties to enrich the related data, which improves our data connectivity to the source’s external database, such as KEGG, UniProt, and the Alliance for glycan-related genes.

In the previous version, each resource was maintained based on its own independent RDF schema that was generated by the data structure of an external database. The factors that led to SPARQL queries returning information on resources were complex and time-consuming. The unified RDF schema enables us to quickly and efficiently provide results from a large database by utilizing metadata of various types. Furthermore, to promote access to glycan information, we support a variety of nomenclatures, including molecular mass and glycan composition, as well as different text formats for glycan nomenclatures: GlycoCT, IUPAC extended, IUPAC condensed, Linear Code, KCF, and WURCS [19]. In particular, the search tool by monosaccharide composition is extremely beneficial for confirming MS data that lacks glycosidic linkage information because it shows which glycans have previously been registered and which have not. The search tool is implemented using GlyTouCan’s advanced technology, which enables the registration of glycans that only contain monosaccharide components without glycosidic bond information, as well as monosaccharides that contain chemical modifications. They are continually developing to accommodate the diverse range of glycans found in unusual organisms and plants.

### Updated resources

#### Genes

**Fig. 3:**
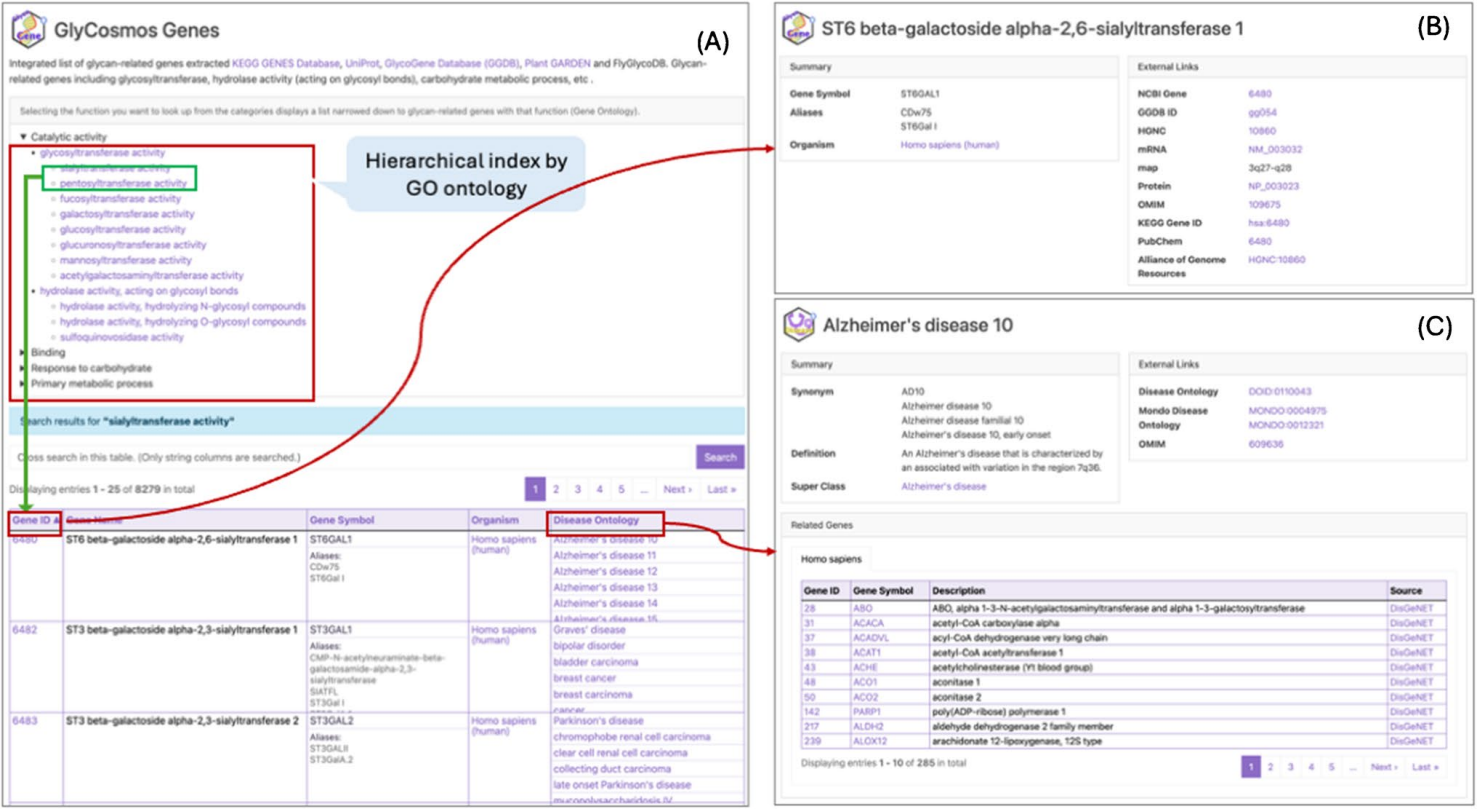
Glycogene interface and individual web page. (**A**) The tree view presents the GO’s hierarchical structure. By searching for GO terms, users can narrow down gene lists associated with specific gene functions. (**B**) Selecting the corresponding gene ID shows an individual page with detailed information, including glycoprotein, ortholog, reaction, expression pattern, and disease. Selecting a prepared content table list provides easy access to glycogene-related information. (**C**) Clicking on a specific disease from the narrowed gene list will open the page for its associated disease

The Genes resource refers to genes that are involved not only in glycosylation but also in the metabolic processes of carbohydrates. They are retrieved from UniProt [20] using the following Gene Ontology (GO) terms, including all of its child terms [21]: “glycosyltransferase activity,” “hydrolase activity, acting on glycosyl bonds,” and “carbohydrate metabolic process.” It is necessary to provide a list of genes that are specifically focused on their functions related to glycans, which includes the ability to search by the exact name of the glycogene. This list will help researchers identify key genes involved in glycan synthesis and modification, facilitating a deeper understanding of their roles in various biological processes. Thus, the Genes entry page was prepared with a hierarchical tree structure based on the GO ontology, allowing users to browse and narrow down the list of gene functions they are interested in. Users can also filter out the results using the dialogue table providing filtering functionality on the left side of the page, allowing searches by specific gene name, gene ID, species name, or disease ontology (Fig. 3A).

Clicking on a Gene ID in the gene table transfers users to the detail page for the gene. Because the gene resource contains a vast amount of information from various sources, the detail page provides an indexed table that enables users to quickly scan the gene-related information and to access the corresponding details (Fig. 3B). Among the related information, the Human Protein Atlas (HPA) illustrates the human tissues in which the gene is highly expressed. The Alliance of Genome Resources (Alliance) [22] section shows the tissues of other species, such as mice, yeast, worms, fruit flies, zebrafish, and rats, in which it has been found to be expressed. The enzymatic information about glycan-related genes involved in the biosynthesis, hydrolysis, and translocation of glycans are integrated from the Rhea database, an ontology-based knowledge base for biochemical reactions [23]. Additionally, the linked KEGG [24] orthology data helps infer the unknown function of genes in other species. The Disease content connecting the gene and disorders displays gene-associated diseases, derived from the integrated data of the GlycoDisease Genes Database (GDGDB) [25] and the Alliance of Genome Resources (Fig. 3C).

#### Glycans

The GlyCosmos Glycans resource provides up-to-date glycan information sourced from GlyTouCan, the international glycan repository [26], with weekly updates. Thanks to the GlyTouCan repository equipped with a validation system that checks the correctness of the WURCS format, which is one of the standards to represent a wide range of glycan structures, including those with ambiguous or incomplete information that is common in glycan structure analysis [27], to issue a unique identification number (ID) to glycans, GlyCosmos Glycans can use these IDs to facilitate the exchange and integration of glycan data. The table of glycan resources displays a GlyTouCan ID with SNFG format, an IUPAC condensed name, a glycan motif, a subsumption level that indicates the degree of detail in the glycan structure, a calculated molecular mass from mass spectrometry, and a species list that includes the taxonomic hierarchy. The individual glycan page provides comprehensive glycan-related information, including tissue localization, lectin-glycan interactions, enzyme reactions involved in glycan construction, and glycan-related pathways. In the latest update to GlyCosmos v4, the 3D structural information has been provided by integration with GlycoNAVI (https://glyconavi.org), GlycoShape (https://glycoshape.org/), and GLyCAM (https://glycam.org) because three-dimensional (3D) structural details are crucial for understanding the function and interactions of glycans in diverse biological systems.

**Fig. 4:**
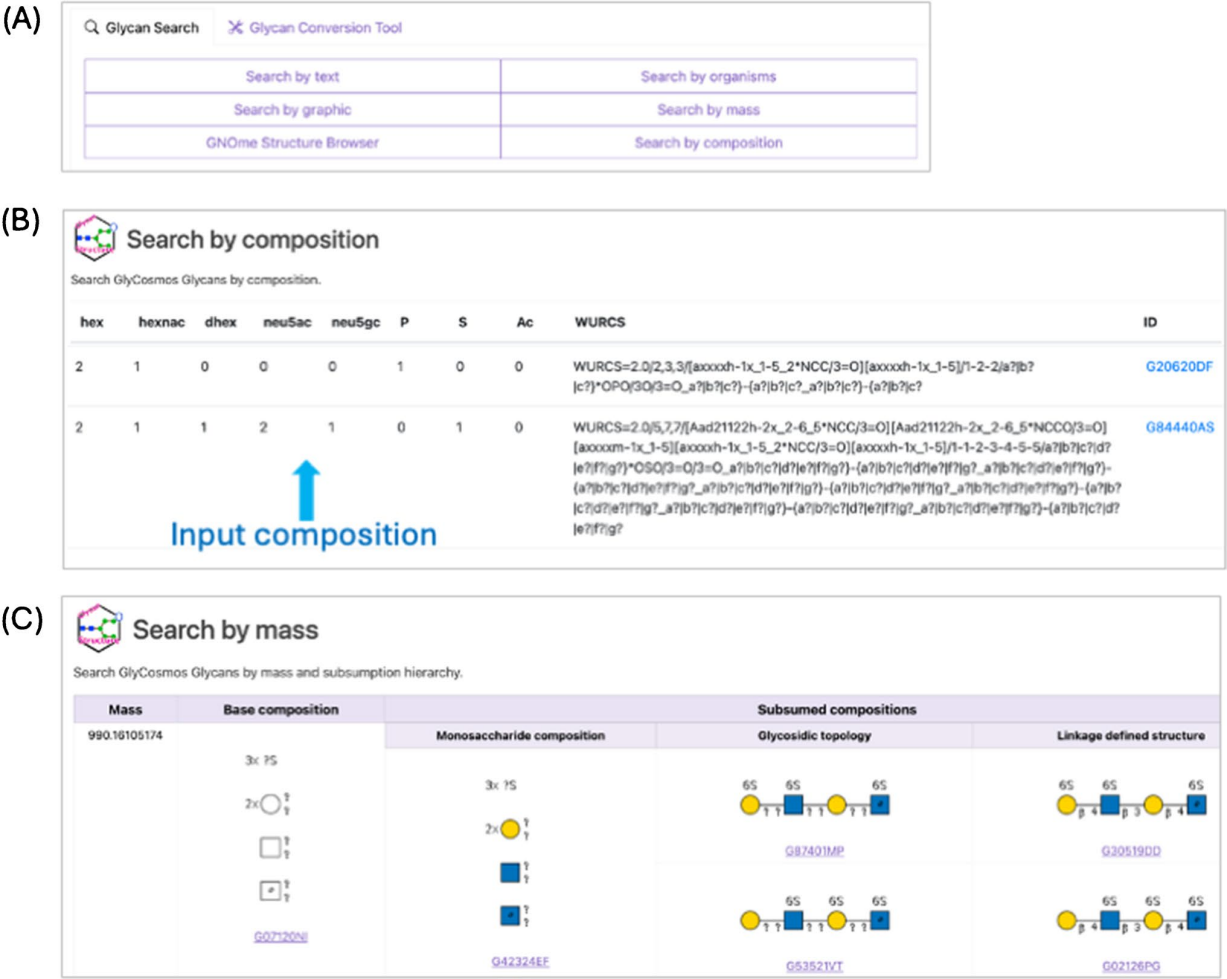
Screenshot of the Glycan Search and results. (**A**) Search types that are supported by our system. (**B**) After the user inputs the composition of a monosaccharide base and its substituents, the system generates a WURCS string and verifies its registration in GlyTouCan. If it is registered, the system provides the corresponding GlyTouCan ID. (**C**) The search by molecular mass and probable range shows the subsumption results in order of glycan structure accuracy

The enhanced search engine offers multiple input options to retrieve glycan information, such as text name of glycan, species, molecular mass, and drawing (Fig. 4A). The text search option supports several of the main formats, including GlycoCT, IUPAC condensed, IUPAC extended, linear code, KCF, and WURCS. The search option based on glycan composition allows users to find glycan structures using only the monosaccharide composition, representing the most minimal information about a whole glycosyl structure. The system has also the option to upload data from Glycomics as a file, which is very useful for handling considerable quantities of data (Fig. 4B). The search field by molecular mass feature allows users to retrieve data based on a specific range of molecular weights. When one enters a molecular mass for MS analysis and its uncertainty range, the table displays a hierarchical ordered list of the matching results. We categorized the results of subsumption based on the level of detail in the information on glycan structure. The classification is presented in order of ambiguity: the base composition specifies the number and types of component monosaccharides, such as two hexoses or one *N*-acetyl hexosamines (HexNAcs); the Monosaccharide composition specifies the number and types of known hexoses, such as mannose or galactose; the Glycosidic topology specifies component monosaccharides of the SNFG symbol level and connectivity between them but not the exact glycosidic bonds; the Linkage defined structure specifies the most detailed level showing a topology between adjacent monosaccharides, including the previous detail level (Fig. 4C). The system of search by mass will be useful to obtain comprehensive information on glycans when only partial information on glycans at different levels is generated during glycomics analysis.

#### Lectins

Glycan arrays are essential tools for examining the glycan-binding specificity of glycan-binding molecules (GBMs) to understand glycan function in interactions between cells and between pathogens and cells. Beginning with the development of the CFG (Consortium for Functional Glycomics) array for mammalian glycans, various glycan arrays have been developed reflecting glycans discovered in microbes [28] and plants [29] thanks to the technical advancements in glycan synthesis. However, it is not easy to find binding specificities between glycans and individual GBMs in thousands of glycan array datasets. The CarboGrove database provides glycan structures bound to lectins based on experimental results from various glycan arrays and lectins sourced from different suppliers [30]. Also, they show glycan motif information based on their own software, the MotifFinder, which aids users in estimating the binding specificity of lectins, leading to low confidence in glycan profiling because of the broad binding specificity of lectins on glycans [31]. UniLectin is another database that provides details on lectins, including their structure, species, and the glycans they bind to. Users can find binding diversity observed between a lectin and various glycan structures containing common glycan motifs [32].

In the initial release of the GlyCosmos Portal, the Lectin resources were organized based on the carbohydrate-binding proteins identified from UniProt [33] and information on lectin-binding glycans was obtained from the Lectin Frontier DataBase (LfDB), which offers quantitative intensity values reflecting binding affinity between lectins and glycans based on frontal affinity chromatography experiments [34]. We have provided the glycans of the three top-ranked binding affinities from LfDB. To reflect the increasing lectin information through the progressive development of the lectin arrays and the growth of its analysis data, the resources from the CarboGrove and UniLectin databases have also been integrated into GlyCosmos.

**Fig. 5:**
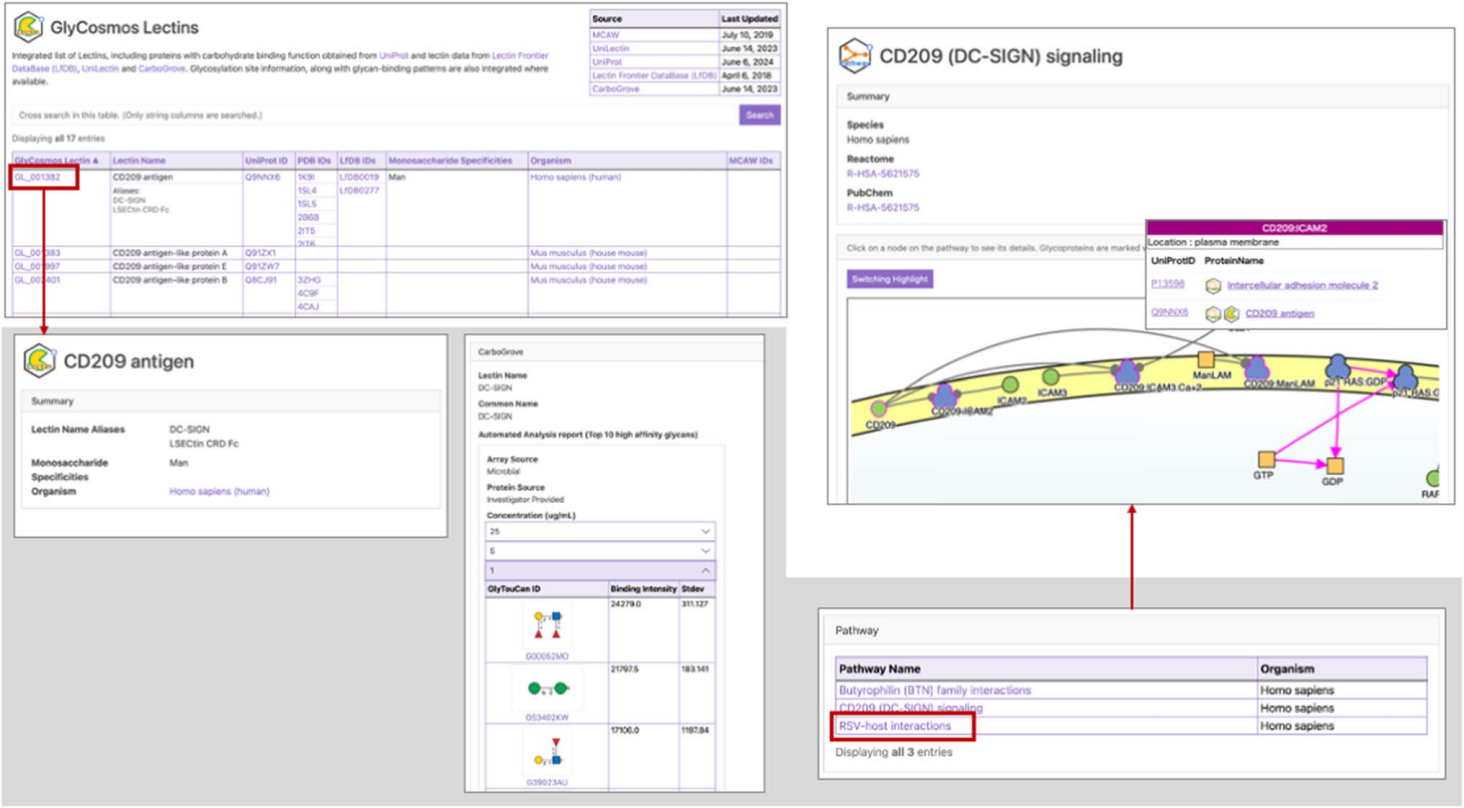
Screenshot of the CD209 lectin protein search. By selecting the lectin ID from the filtered result table using different metadata, users can access the detailed page for the lectin. This page provides information about the binding specificity of the glycan based on the concentration of the lectin, as provided by the CarboGrove database, and accesses pathway information that shows the role of the lectin protein within the cell. The Lectin resource is enclosed in a gray box. Clicking the linked pathway information opens the pathway resource box in the upper right corner

This integration enables users to obtain extensive information related to lectins. In the GlyCosmos Lectins table, users can browse through the available data and sort the results by clicking the resources they are interested in, such as species, recognized monosaccharide, or the name of the lectin. Clicking a Lectin ID will open its detail page with more detailed information. For example, DC-SIGN (CD209, CLEC4L) is one of the lectins showing the broadest specificity across several species. By using the lectin name in the filtering tool to aid in the search process, a sorted table that includes the lectin name of “CD209 antigen” is displayed, and subsequently, this result table can be reorganized according to selected metadata, such as species or UniProt ID. Choosing the lectin ID specific to the human species gives access to a detailed page that provides GO terms, allowing us to inspect the molecular function or biological process, such as peptide antigen binding or fucose binding for pathogenic microbes, in which DC-SIGN lectins are involved [35]. Through the integration of the Reactome database, users can access the pathway data that CD209 has been involved in, which enables us to investigate the role of this lectin. In particular, integrating CarboGrove allows us to identify the binding specificity of the partner glycans against a lectin with the ten glycan structures of high affinity according to lectin concentration, helping researchers choose a reasonable pair of lectin and glycan. Also, users can see the 3D structure of lectins in the Protein Data Bank (PDB), which helps understand the binding affinity of the target glycan (Fig. 5). Furthermore, by accessing the Glycoproteins resource with the lectin ID, users can obtain the expression of the lectin of interest in tissues or the associated disease information, which is imported from the Human Proteome Atlas (HPA).

#### Diseases

The previous version of the disease resource simply offered a list of diseases related to glycans. To enhance the usefulness of the information, it was important to include details regarding the genes and proteins associated with the disease. The updated RDF schema enables users to search for relevant data between both the disease and gene resources. This integration allows for a more comprehensive understanding of how specific genes and proteins contribute to various diseases, facilitating better research and insights. Consequently, users can easily navigate the relationships between glycans and diseases and their genetic variations.

Since it is known that genetic defects in glycoenzymes for the synthesis of glycans can lead to congenital disorders of glycosylation (CDGs), many studies have revealed the key roles played by glycans in human diseases, such as inflammatory diseases, autoimmune diseases such as rheumatoid arthritis, and cancer [5]. To provide information on diseases associated with glycans, the Diseases resource was prepared by integrating data on diseases and their causal genes from GDGDB in the initial release version of GlyCosmos. GDGDB uses its own ontology, GGDonto, to represent information in a standardized format. From there, we collected 120 hereditary metabolic diseases. These include genes, proteins, and disease annotations from Online Mendelian Inheritance in Man (OMIM) [36] in lysosomal storage disorders (LSDs) and CDGs that are caused by changes in enzymes that make or break down certain glycans.

Over the past years, genome-wide association studies (GWAS) have revealed genetic variants associated with traits influenced by environmental pressures and disease, although this does not imply causal relationships [37].

Additionally, genetic disorders, which are the genes responsible for human disease, have been explored in a range of animal models. DisGeNET provides integrated data from curated databases, such as the GWAS Catalog, the Rat Genome Database (RGD), the Mouse Genome Database (EGD), and the Genetic Association Database (GAD), as well as text-mined data from a wide range of scientific articles in a standardized format. They allow users to explore genes, including non-coding genes, and genetic variations that influence disease risk, such as SNPs, related to a wide range of human diseases. The Alliance provides genes, variants, gene function, and related diseases integrated from databases of six model organisms: budding yeast (*Saccharomyces cerevisiae*), worm (*Caenorhabditis elegans*), fruit fly (*Drosophila melanogaster*), zebrafish (*Danio rerio*), mouse (*Mus* sp.), and rat (*Rattus norvegicus*) [22]. They are developed to comply with the principles of FAIR-ness, which involve the use of ontologies such as GO and DO to describe disease-related resources such as gene functions, processes, and human orthologs.

**Fig. 6:**
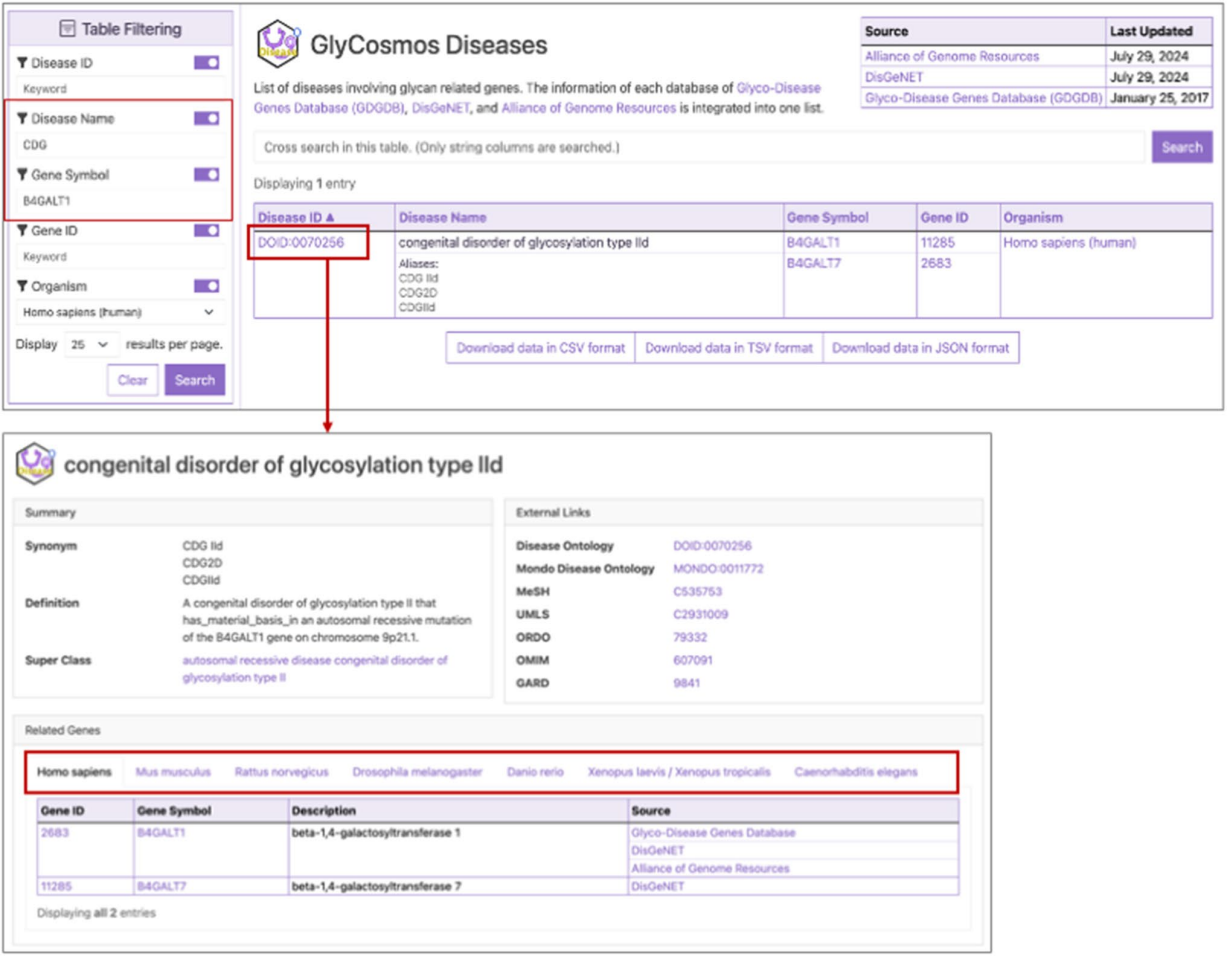
Screenshot of the Diseases resource. The Table Filtering feature allows search results to be reduced by gene names, gene symbols, disease names with a variety of synonyms, and ID. Clicking on a disease ID displays a controlled vocabulary and ontology, developed to unify the diverse terms for disease description in the biomedical domain, such as MeSH, OMIM, and disease ontology, etc.

To keep up with the rapidly growing information on glycan-related diseases in humans and model organisms, GlyCosmos has imported the glycogene-associated disease data from DisGeNET and the Alliance. The Diseases resource is organized using DO, which was developed to provide consistency in terms of the description of diseases and biomedical languages used in diagnosis or therapy. By clicking a disease ID on the list table, users can access detailed information such as synonyms, definitions, and links to OMIM, which demonstrate the relationships between human diseases and genetic phenotypes, as well as the pertinent glycan-related genes. When searching for a certain disease that involves a particular glycogene, a filtering search allows the glycan-related disease results to be narrowed down. For instance, to find the CDG associated with the B4GALT1 (beta-1,4-galactosyltransferase 1) gene, which appears to cause a wide range of physical and developmental abnormalities, enter “b4galt1” in the gene symbol field and “CDG” in the disease name field of the filtering box (Fig. 6). When a disease ID of the result table is selected for a human species, users can access ortholog information from the Alliance and authoritative external references that are standard for disease annotation, such as MeSH, OMIM, Orphanet [38], and Rare Diseases Information Center (GARD) [39] for rare diseases, which are curated by specialists. Also, users can access human phenotypic information linked to a glycogene through the Human Phenotype Ontology (HPO), which is a standardized vocabulary to represent the various clinical symptoms and manifestations associated with genetic disorders and diseases [40].

#### Pathways

Pathway data provides an integrated view of the biological process, including gene regulation or signaling and metabolism, which shows the relationship between the genomic regions and their products in a cell, tissue, or organism under different conditions. GlyCosmos Pathways has provided the glycan-related pathway data through mapping a GlyTouCan ID or Uniprot ID of glycoprotein to the Reactome [41], which is a manually curated and peer-reviewed database that provides the tools for pathway analysis and pathway information with biomolecule details. The glycan-related pathway data is obtained by mapping it with the protein ID in UniProt for the 16 species. The mapped data is provided in a standard format called BioPAX, which is developed by the community to facilitate the exchange or sharing of pathway data [42]. However, in terms of retrieving query results, in order to access a glycan or protein resource node from a pathway node, it is necessary to traverse multiple paths that connect all the resource nodes, such as reaction, pathway step, catalysis, and so on, that are involved in the pathway description. This resulted in the need for complex SPARQL queries for searching, which in turn caused a delay in the retrieval of results. To address this issue, we designed to directly connect the glycan and protein resources to the Pathway resources using the http://semanticscience.org/resource/SIO_000128 (is contained in) property of the SIO ontology in the new RDF schema (Fig. 2).

**Fig. 7:**
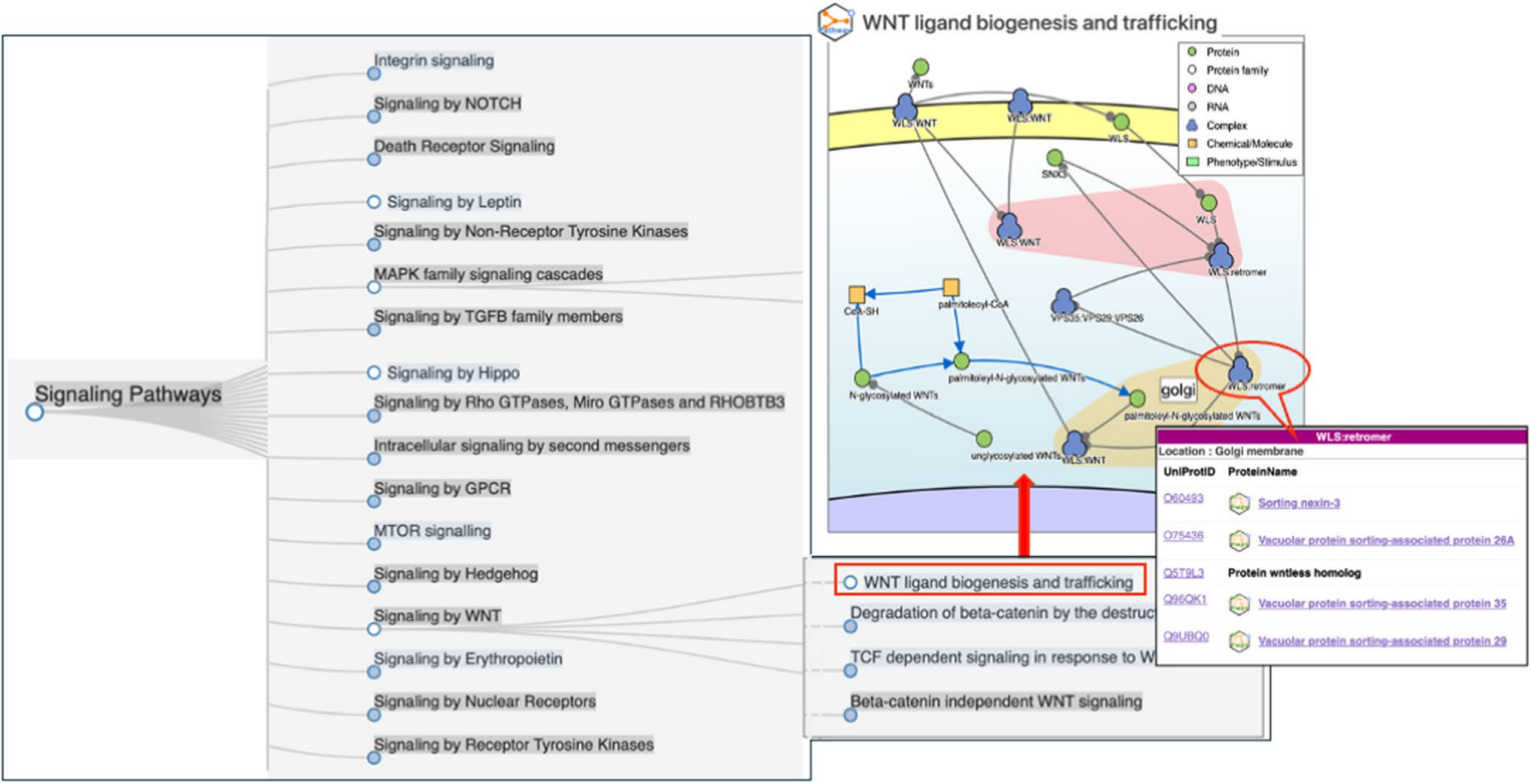
The glycan-related pathway data. The pathways associated with glycans and glycoproteins are represented in a tree structure by inference. To display a pathway, simply click on the empty circle located in front of the pathway name. By selecting the protein of interest in the pathway diagram, users can ascertain its intracellular location and identify whether it is a glycosylated protein or a non-glycoprotein, as indicated by the logo implying whether or not it is a glycan-related resource

Like the Gene Ontology (GO), the pathway data of Reactome are also organized in a hierarchical structure. We leveraged this feature to enable users to look into the organized pathway list based on ontology, including that corresponding pathway. Using an inference function through a SPARQL query, the pathways that have parent–child relationships can be retrieved, even if they are not directly connected to the queried glycans or glycosylated proteins. Thus, users are able to inspect a wider range of pathways that involve glycan-related resources, along with a hierarchical tree structure (Fig. 7). This will be useful because the pathway lists allow users to search for the pathway information by a pathway name without requiring knowledge of the hierarchical structure of the pathway that is specified by the developers.

## Discussion

The GlyCosmos Portal is a publicly accessible database that has been continuously updated and incorporated with various omics data since its initial release. We are also continuously developing new tools and incorporating more resources, such as GlycoStore [43], which allows users to conveniently access information on GU (glucose units), AU (arabinose units), and retention time of HPLC and m/z of MS data. Along with the data from GlycoBase [44] and GALAXY (https://glycosmos.org/galaxy), all the referenced glycans in these resources have assigned GlyTouCan IDs, thus allowing us to integrate this information with other available omics data. In the future, we plan on creating an integrated LC data resource combining these resources.

There are a few ways that we have made an effort to integrate data. One is to promote the sharing of data within the community. To share data and collect the latest results from glycan-related research, the GlyCosmos Portal has established important repositories that ensure long-term preservation of data, alleviating researchers from the burden of keeping and backing up their data. These repositories further enhance the benefits that users obtain from integrated datasets. We are now working on establishing a pipeline that will automate the process of assigning GlyTouCan IDs to MS peaks and raw data derived from glycoproteomics and glycomic analyses.

Also, we have endeavored to improve the functionality of GlyTouCan, the international glycan repository, to establish its reliability, which will aid in better communication between databases when they reference complex glycan structures. Thanks to GlyTouCan’s enhanced function that validates the glycan to be registered, the Glycans resource of GlyCosmos can provide users with validated glycans that ensure chemically accurate glycan structure. Also, it permits glycans to be linked to the main resources, enabling users to infer glycans’ roles in various biological processes. Similar to GlyTouCan, the development of a system that can verify the accuracy of various molecules, including lipids, peptides, and proteins, will enhance the authenticity of registered data, which will encourage researchers to engage in data sharing. Furthermore, in terms of data sharing, it might be helpful to build a system in which the community provides researchers sharing their data with an incentive comparable to publication. Although it is still challenging to find and integrate a diverse range of tremendous datasets that might be pertinent to glycans, we expect that the GlyCosmos repositories, which can accommodate a wide range of resources, will fill the gap.

The other way is to standardize data. To optimize data sharing and reuse, we have complied with semantic standards, such as RDF, SPARQL, and ontologies, and implemented validation procedures for the reliability of data. These standards have provided a way to resolve the lack of consistency in data formats across diverse datasets and to accurately describe the resources. Thus, the GlyCosmos Portal can capture the connections between genes, proteins, pathways, diseases, and other entities, and it can offer insights that arise from these connections. Furthermore, ontologies are essential to encompass expanding datasets, such as experimental results from genetic or epigenetic change and glycan interactome studies, establish relationships between these results and glycans, and support the precise interpretation of these relationships. Specifically, the ontology for knowledge representation enables the discovery of new knowledge due to its computational inference, as demonstrated by the example of leveraging the GO ontology, which will accelerate the integration of intricate biological data and result in the creation of a knowledge graph capable of addressing complicated questions. Moreover, we have developed a new RDF schema to enhance the interconnectivity of resources that are disparately developed on their own schemas and vocabularies.

The last is to develop tools that will aid in the analysis of glycan-related information from various aspects. Several useful tools are categorized in the Tools section: GlycoMaple helps users inspect glycan-related metabolic pathways using RNA-seq data; GlycanBuilder, a glycan drawing tool, simplifies the process of creating a glycan in SNFG format and simplifies the registration process by offering a search function for checking whether it is already registered in GlyTouCan; and MCAW allows multiple glycans to be aligned simultaneously, revealing the glycan substructures with the highest binding affinity to glycan-binding proteins. GlycanBuilder also allows users to easily obtain image files for the glycans they draw in it.

Despite the efforts to collect and integrate data, there are still gaps in understanding the role of glycans in a variety of biological processes because of the remaining data in scientific databases and the literature. We expect that glycomics data can be integrated with other omics data through the development of simple and efficient systems and the cooperation of researchers in the life and medical sciences. We anticipate that GlyCosmos will serve as a solid foundation for the discovery of new knowledge while also promoting awareness among scientific communities about data sharing.

## Data Availability

The GlyCosmos data is available at the following URL: https://glycosmos.org/download.
